# Can levels of antioxidants in synovial fluid predict the severity of primary knee osteoarthritis: a preliminary study

**DOI:** 10.1186/2193-1801-2-652

**Published:** 2013-12-05

**Authors:** Chayanin Angthong, Noppawan Phumala Morales, Werasak Sutipornpalangkul, Anuwat Khadsongkram, Piya Pinsornsak, Boonchana Pongcharoen

**Affiliations:** Department of Orthopaedic Surgery, Faculty of Medicine, Thammasat University, Pathum Thani, Thailand; Department of Pharmacology, Faculty of Science, Mahidol University, Bangkok, Thailand; Department of Orthopaedic Surgery, Faculty of Medicine Siriraj Hospital, Mahidol University, Bangkok, Thailand

**Keywords:** Knee osteoarthritis, Antioxidant, Oxidative stress, Synovial fluid, Prediction

## Abstract

**Background:**

Little is known about differences in amounts of antioxidants or oxidative stress at different stages of knee osteoarthritis. This study investigated the relationship between concentrations of antioxidants, iron and lipid peroxidation in synovial fluid and levels of severity of primary knee osteoarthritis.

**Materials and methods:**

From 2011 to 2013, 23 patients (mean age, 66.7 ± 7.6 years) with primary knee osteoarthritis were recruited. Patients were divided into 2 groups based on pre-treatment knee society scores (KSS): *n* = 9, severe KSS ≤46; and *n* = 14, mild-moderate KSS >46. Synovial fluid was analyzed to determine levels of antioxidants, iron concentrations and lipid peroxidation (thiobarbituric acid reactive substances [TBARs]). Baseline data, including Kellgren- Lawrence radiographic grade, were collected for all patients.

**Results:**

Mean KSS was 49.1 ± 10.8. Total mean concentrations of antioxidants were 2.29 ± 1.71 ng/mL vitamin E and 0.47 ± 0.51 nmol/mL glutathione (GSH). Total mean levels of TBARs and iron were 1.20 ± 0.37 nmol/mL and 2.13 ± 0.82 μg/mL, respectively. The mean concentration of vitamin E was inversely related to severity of knee osteoarthritis (mild-moderate > severe, *p =* 0.006). There were no significant differences between the two groups in terms of GSH (*p* = 0.90), TBARs (*p* = 0.84) or iron levels (*p* = 0.27). There was a significant positive correlation between KSS and vitamin E concentration (*r =* 0.43*, p* = 0.04). No significant correlations were shown between KSS and GSH (*r = -*0.01*, p* = 0.97), TBARs (*r = -*0.06*, p* = 0.81) or iron level (*r =* 0.28*, p* = 0.20).

**Conclusion:**

Using synovial fluid profiles, vitamin E concentration is an essential prognostic factor in primary knee osteoarthritis and may act as a basis for treatment directions. The concentration of vitamin E decreased as the clinical severity of primary knee osteoarthritis increased.

## Introduction

Primary knee osteoarthritis is a major burden in the increasing elderly population. There are several modalities that may play a role as conservative management for knee osteoarthritis, such as glucosamine, diacerine, and viscoelastic injection. In addition, some researchers have reported molecular targeting therapy, such as vitamin E and glutathione, in the role of the antioxidants to treat knee osteoarthritis (Machtey and Ouaknine 1978; Blankenhorn 1986; Jordan et al. 2004). However, some reports have argued against these therapies in the treatment of knee osteoarthritis (Wluka et al. 2002; Brand et al. 2001), and these treatments remain controversial (Brand et al. 2001). Although a previous study has shown that vitamin E levels were significantly lower in the synovial fluid of patients with primary knee osteoarthritis compared with patients with knee injury (Sutipornpalangkul et al. 2009), little is known about differences in the amounts of the antioxidants or oxidative stress at different stages of primary knee osteoarthritis.

The present study set out to investigate the relationship between concentrations of antioxidants, iron, and lipid peroxidation in synovial fluid and levels of severity of primary knee osteoarthritis. The results of this study may help in the treatment of primary knee osteoarthritis in the future.

## Material and methods

From February 2011 to February 2013, patients with primary knee osteoarthritis (Jordan et al. 2004; Sutipornpalangkul et al. 2009) were recruited to participate in the study, following approval from the institutional ethics committee. Inclusion criteria were patients with primary knee osteoarthritis and age > 55 years. Exclusion criteria were patients with rheumatoid arthritis or secondary osteoarthritis (such as post-traumatic, post-infection osteoarthritis), concomitant knee or adjacent area infection, allergy to viscoelastic medication (injection), post-steroid injection, post-knee arthroplasty, immunocompromised patient, poor nutrition, severe knee pain (from any cause in addition to primary knee osteoarthritis) with normal radiographic findings, and poor medical condition. All 24 patients were consecutively enrolled according to the inclusion and exclusion criteria. One male patient was excluded because his age was less than 45 years, leaving 23 patients in the present study. All 23 patients were consecutively enrolled according to the inclusion and exclusion criteria. Three patients were from the outpatient clinic, where they provided synovial fluid (1 mL) from arthrocentesis under sterile technique before viscoelastic injection treatment. The other 20 patients provided synovial fluid from surgery (knee arthroplasty). Surgeons retrieved synovial fluid (1 mL) after knee arthrotomy, at the beginning of the operation before bone reaming, cutting, or implantation. Our preliminary study showed that mean knee society score (KSS) (Insall et al. 1989; Asif and Choon 2005) of patients with knee arthroplasty was ~46. Therefore, we decided to divided all 23 patients into 2 groups based on pre-treatment (viscoelastic injection or knee arthroplasty) KSS (Insall et al. 1989; Asif and Choon 2005): ≤46, severe; and >46, mild-moderate. Baseline data, including Kellgren- Lawrence (KL) radiographic grade (Kellgren and Lawrence 1957), were collected from all patients. This study was approved by the ethic committee of the Faculty of Medicine, Thammasat University.

### Synovial fluid analyses

Synovial fluid from each patient was centrifuged at 2330 × *g* (800 RPM) for 5 minutes. The supernatant was collected to assay levels of antioxidants, including α-tocopherol (vitamin E) and total glutathione (GSH), iron concentrations and lipid peroxidation (TBARs) using the following techniques.

### Determination of lipid peroxidation (TBARs) levels

TBARs levels were measured fluorometrically using a spectrofluorometer (Perkin-Elmer LS55 luminescence spectrometer, Beaconsfield, UK) with excitation and emission wavelengths at 515 and 553 nm, respectively. 1,1,3,3-tetraethoxypropane was used as the standard (Asakawa and Matsushita 1980).

### High performance liquid chromatography (HPLC) determination of α-tocopherol

Levels of α-tocopherol (vitamin E) in synovial fluid were determined by reverse phase HPLC following a modified method by Zaspel and Csallany (1983). Two hundred and fifty microliters of samples were sequentially extracted with methanol and hexane. After drying the hexane layer under N_2_ and re-dissolving with methanol, the extract was injected into the HPLC system, which consisted of a Waters 2695 (Waters, Milford, MA, US). Vitamin E levels were determined with a Jusco FP 2020 Plus fluorescence detector (Japan Spectroscopic Co. Ltd, Tokyo, Japan), with an excitation wavelength of 295 nm and emission wavelength of 370 nm. The software program, Empower Pro (Waters, Milford, MA, US), was used for data analysis. The separation was carried out on a Nova Pak C18 column (4.6 mm · 150 mm, 5 μ) with 100% methanol as the mobile phase. The flow rate was 1.0 mL/min.

### Determination of GSH levels

Total GSH in synovial fluid samples was determined using the DTNB-glutathione reductase recycling method with 5, 5′ dithiobis 2-nitrobenzoic acid as the disulfide chromogen. The absorbance of the reduced chromogen was measured with a microplate reader at 405 nm (Biotex, USA) and GSH concentration was expressed as nmol/mL (Anderson 1985).

### Determination of total iron levels

Total iron in synovial fluid was determined using a modification of the methods of Foy et al., and ferrozine (Sigma, St. Louis, MO, US) was used as a chromogen (Foy et al. 1967). An aliquot of 250 μL of synovial fluid was mixed with 500 μL of acid mixture (10% trichloroacetic acid, 1 mol/L hydrochloric acid), and boiled for 30 min. After cooling, the samples were centrifuged at 2330 × *g* for 10 min. The clear supernatant was then mixed with the chromogen (1.5 mol sodium acetate and 0.5 mmol BPT) in 1:1.5 v/v and left at room temperature for 20 min before reading the absorbance at 562 nm with a UV-visible spectrophotometer (Cintra 10e, GBC, Melbourne, VIC, Australia).

### Statistical analyses

Statistical analysis was implemented using SPSS software version 13.0 (SPSS Inc., Chicago, IL, USA). ANOVA was used to analyze the statistical significance of differences in the values of concentrations of vitamin E, GSH, iron or TBARs between the 2 different groups of knee osteoarthritis severity. The correlations between the KSS and the concentration of vitamin E, GSH, iron or TBARs were analyzed and interpreted via Pearson’s correlation coefficient (*r*). Categorical variables were analyzed using Fisher’s exact test or Chi-square test. The level of significance was identified at *p* < 0.05.

## Results

The patient population comprised 18 females and 5 males. The mean age was 66.7 ± 7.6 years (range: 55–86). Mean knee society score was 49.1 ± 10.8 (range: 23–70). Patients were divided into 2 groups based on KSS: *n* = 9 (severe: KSS ≤46); and *n* = 14 (mild-moderate: KSS >46) (Table 1). There were no significant differences in mean age (*p* = 0.67) and body mass index (*p* = 0.16) between the two groups (Table 1). No significant difference was found in the distribution of genders between the two groups (*p* = 0.12) (Table 1). In terms of the radiographic findings of the 2 groups, the distributions for the severe group were as follows: *n* = 9 patients (KL-IV: 5; KL-III: 4); for the mild-moderate group, *n* = 14 patients (KL-IV: 8; KL-III: 4; KL-II: 2) (*p* = 0.47).

**Table 1:** **Demographic data of patients in all groups**

		Mild-moderate group	Severe group	***P***-value
		(KSS >46)	(KSS ≤46)	
		***n*** = 14	***n*** = 9	
Age^a^ (yrs) (range)		66.14 ± 7.25 (55–78)	67.56 ± 8.47 (55–86)	0.67
Gender				0.12
	- Female	9 (64.30%)	9 (100%)	
	- Male	5 (35.70%)	0 (0%)	
Body mass index^a^ (range)		24.63 ± 3.53 (20.00–30.80)	26.76 ± 2.88 (23.86–32.42)	0.16

^a^Mean ± standard deviation (S.D.). *Abbreviation*: *KSS* Knee society score (Insall et al. 1989; Asif and Choon 2005), *yrs* years.

### Synovial fluid analyses

Total mean concentrations of antioxidants were 2.29 ± 1.71 ng/mL vitamin E and 0.47 ± 0.51 nmol/mL GSH. Total mean levels of TBARs and iron were 1.20 ± 0.37 nmol/mL and 2.13 ± 0.82 μg/mL, respectively. We found that only mean concentrations of vitamin E were significantly different between the two groups (mild-moderate > severe: *p* = 0.006) (Table 2). There were no significant differences between the two groups in terms of GSH (*p* = 0.90), TBARs (*p* = 0.84), or iron concentration (*p* = 0.27). There was a significant positive correlation between KSS and the concentration of vitamin E (*r =* 0.43*, p* = 0.04). No significant correlations were shown between KSS and GSH (*r = -*0.01*, p* = 0.97), TBARs (*r = -*0.06*, p* = 0.81), or iron levels (*r =* 0.28*, p* = 0.20).

**Table 2:** **The concentrations of vitamin E, glutathione, iron and TBARs of the patients in all groups**

	Mild-moderate group	Severe group	***P***-value
	(KSS >46)	(KSS ≤46)	
	***n*** = 14	***n*** = 9	
Vitamin E^a^ (ng/mL) (range)	3.03 ± 1.75 (0.96–6.50)	1.12 ± 0.74 (0.00–2.10)	0.006*
Glutathione (nmol/mL) (range)	0.48 ± 0.55 (0.00–2.00)	0.45 ± 0.48 (0.00–1.63)	0.90
Iron (μg/mL) (range)	2.28 ± 0.99 (1.28–5.29)	1.89 ± 0.42 (1.43–2.69)	0.27
TBARs^a^ (nmol/mL) (range)	1.22 ± 0.44 (0.76–2.28)	1.18 ± 0.23 (0.79–1.60)	0.84

^a^Mean ± standard deviation (S.D.). *Abbreviation*: *KSS* Knee society score (Insall et al. 1989; Asif and Choon 2005), *TBARs* Thiobarbituric acid reactive substances. *significant difference.

## Discussion

Sutipornpalangkul et al., have shown that vitamin E levels were significantly lower in the synovial fluid of patients with primary knee osteoarthritis compared with patients with knee injury (Sutipornpalangkul et al. 2009). However, it may be difficult to know the real differences in the amounts of antioxidants or oxidative stress at different stages of knee osteoarthritis because of a lack of previous studies reporting subgroup analyses of patients with various levels of severity. This point led us to design the present study to investigate the relationship between concentrations of vitamin E, GSH, iron, and lipid peroxidation in the synovial fluid and levels of severity of primary knee osteoarthritis. The results of our study may help to clarify the role of antioxidants and oxidative stress in the patients with various severities of primary knee osteoarthritis.

We found that only the concentration of vitamin E was significantly different between mild-moderate and severe primary knee osteoarthritis according to the patients’ clinical features. However, there were no significant differences between the two groups in terms of GSH, TBARs, iron levels or KL grades. For the non-significant difference between the two groups in KL grades, the histological evaluation of the articular cartilage may be more useful than KSS to set the severity of osteoarthritis. In addition, a significant positive correlation between KSS and concentrations of vitamin E (*r =* 0.43*, p* = 0.04) was observed. There were no significant correlations between KSS and GSH (*r = -*0.01*, p* = 0.97), TBARs (*r = -*0.06*, p* = 0.81), or iron levels (*r =* 0.28*, p* = 0.20). Our analyses show that vitamin E is a crucial factor for predicting the severity of primary knee osteoarthritis. The concentration of vitamin E decreased as the severity of primary knee osteoarthritis increased. The levels of GSH, lipid peroxidation via TBARs expression, and iron concentrations did not show associations with the severity of primary knee osteoarthritis. These findings confirm the results of previous studies that showed synovial fluid from subjects with knee osteoarthritis was characterized by significantly decreased antioxidant levels compared with the reference group of knee joints with intact cartilage (Sutipornpalangkul et al. 2009; Regan et al. 2008). Our report may be a latest study, which demonstrated a reversed trend of vitamin E concentration and severity of primary knee osteoarthritis. The role of vitamin E as an antioxidant to treat ongoing knee osteoarthritis has been reported previously (Machtey and Ouaknine 1978; Blankenhorn 1986; Jordan et al. 2004). Bhattacharya et al. also reported the protective role of vitamin E supplementation against oxidative stress-mediated biomolecular worsening of knee osteoarthritis (Bhattacharya et al. 2012). In addition to vitamin E, other disturbances of oxidative equilibrium, such as the activities of catalase and glutathione peroxidase, were found in patients with knee or hip osteoarthritis (Olszewska-Słonina et al. 2010). Future treatments may be developed based on this knowledge. However, reports of the efficacy of treatment with vitamin E supplementation at different levels of severity of primary knee osteoarthritis are still lacking. Our findings may help clarify the prominent role of vitamin E in primary knee osteoarthritis with different severity. Further large-scale studies of the influence of vitamin E supplementation in primary knee osteoarthritis with different levels of severity are needed based on the findings of the present study.

The current study had some limitations. This report derived from our preliminary study, which had low numbers of patients with severe or mild-moderate severity of knee osteoarthritis. These limited numbers of patients may not be able to reveal significant differences in some parameters, such as GSH, iron, and lipid peroxidation. A further limitation of this study is the lack of models of univariate regression analysis considering KL and/or histological grades as independent variables to sufficiently correlate vitamin E, GSH and TBARs levels with the severity of osteoarthritis. However, we believe the preliminary findings of our study as to the prognostic value of vitamin E in synovial fluid might offer new treatment directions, such as higher doses of vitamin E supplementation with higher severity of primary knee osteoarthritis.

## Conclusion

Using synovial fluid profiles, vitamin E concentration is an essential prognostic factor in primary knee osteoarthritis and may act as a basis for treatment directions. The concentration of vitamin E significantly decreased as the severity of primary knee osteoarthritis, as measured by the KSS, increased. Our findings may help to clarify the prognostic value of vitamin E in primary knee osteoarthritis with different levels of severity.
